# Advances in Nutrition: Opportunities and Challenges in 2022

**DOI:** 10.3390/nu15102282

**Published:** 2023-05-12

**Authors:** Francisco J. Pérez-Cano

**Affiliations:** 1 Department of Biochemistry and Physiology, Faculty of Pharmacy and Food Science, University of Barcelona, 08028 Barcelona, Spain; franciscoperez@ub.edu; Tel.: +34-9-340-24505; 2 Nutrition and Food Safety Research Institute (INSA-UB), 08921 Santa Coloma de Gramenet, Spain

Many aspects of how food and diet can improve individual health, performance, and wellbeing remain to be discovered. It is necessary to know the exact composition and nutrient availability of diets (food types, preparation, combinations, etc.), the characteristics of individuals (genetics, microbiome, physiological or pathophysiological status, age, etc.), as well as those of the environment that surrounds them. In this sense, this Special Issue entitled “Advances in Nutrition: Opportunities and Challenges in 2022”, edited by Prof. Dr. Philip J. Atherton, Prof. Dr. Ina Bergheim, Prof. Dr. M. Luisa Bonet, Prof. Dr. David C. Nieman, and Dr. Francisco J. Pérez-Cano, has focused on aspects of nutrition and nutritional sciences that are already at the center of current concerns, and which may help humans to face envisaged social and health scenarios in the coming years. The research into nutrition has evolved remarkably in recent years, mostly by applying post-genomic technologies to ascertain mechanisms of nutrient action, including biomarkers of nutrition and health and precision nutrition.

The first aspect to highlight in this Special Issue is the diet–disease relationship. In this sense, metabolic alterations such as diabetes mellitus (DM) and obesity, among others, have in recent years become the most prevalent non-communicable diseases, which in turn provokes a high health-associated cost. For that, there is increasing interest in finding preventive and therapeutic solutions. The review article of Correia et al. [1], as part of a larger project (PARTNERSHIP, Putting the pAtient fiRsT: maNagemEnt of chRonic diSeases by tHerapeutIc Patient education focused on the management of chronic disorders), consisted of a systematic review and meta-analysis of the efficacy of therapeutic patient education (TPE) interventions for DM and obesity on a wide number of biomedical, psychosocial, and psychological outcomes. Among the expected biomedical findings, such as the evident effectiveness of TPE interventions in improving serum HbA1c levels in DM patients, is noteworthy that these preventive actions seem not to be guided by multidisciplinary teams, in which the nutritionist would play a key role.

Second, besides the role of nutrition on particular diseases, of importance is the life period of the study, especially if the individual has an opportunity for improvement. Thus, nutrition is critical during pregnancy for both the mother’s and the child’s health and their short/long-term outcomes. The article of Lisso et al. [2] included in this Special Issue evaluated the adherence to nutritional recommendations in Italy during the 3 pregnancy trimesters in 176 women from normal weight and overweight conditions. Overall, the authors found that independently of their weight during pregnancy, Italian women from the study did not adhere to nutritional recommendations, having a lower caloric and certain micronutrients intake but higher protein and sugar intakes. These results, in line with other studies, highlight the importance of providing adequate counseling, including both educational and supplementation interventions if required, to ensure optimal maternal metabolism and fetal growth and development during pregnancy.

Third, in addition to the impact that dietary modifications can have on health and disease management, the use of dietary supplements should be also considered. This Special Issue includes two different in vivo approaches studying dietary supplementations, their impact on microbiota compositions, and also their impact on certain health conditions. On the one hand, as gut microbiota can play a critical role in the progression of Alzheimer disease (AD), Rossell-Cardona et al. [3] have studied whether the neuroprotective effects of a particular supplement, the spray-dried porcine plasma (SDP), involve the microbiota–gut–brain axis (GBA). The authors found that the dietary supplementation with SDP induced a new intestinal microbial balance that seemed to reduce the intestinal permeability and local and systemic immune and inflammatory responses, which may be involved in the neuroinflammation process of the cognitive decline in these senescent mice model studied. On the other hand, Kienesberger et al. [4] compared the activity of several probiotics with their derived postbiotics, defined as the preparation of inanimate microorganisms and/or their components that confer a health benefit on their host functionality. The authors found that the postbiotic supernatants had some in vitro antibacterial and antifungal effect and modulated the composition of BALB/c mice microbiota. Both the SDP and the postbiotics assayed are then new products that can exert their actions indirectly through microbiota modulation, thus reinforcing interest in these types of supplements. Further studies in this line to gain a deeper insight into the mechanisms but also to prove their effect on humans are needed in order to guarantee their benefit.

Finally, new biomarkers and methods are appearing to connect nutrition and genotype/phenotype. In this sense, the expression of microRNA (miRNAs) is related to certain pathological conditions, such as asthma. It is plausible that these changes could be influenced by certain nutrients or bioactive compounds. Taken all together, Castro et al. [5] found some association between the dietary acid load and those miRNAs related to asthma in the exhaled breath condensate of 150 school participants. The results from this interesting approach should be confirmed in future studies.

In summary, in the study of the relationship of food, dietary patterns, and health, some other aspects should be considered. On the one hand, the interaction of diet and genetics, microbiota, age, or lifestyle, such as exercise and performance, is critical. However, on the other hand, the relationship between nutrition, immunity, and infection has to be considered. In addition, many new food sources and products are or could be on the market, and they deserve to be well characterized in terms of their mechanisms of action and their impact on human health. Finally, any new approaches used for identifying or describing the biomarkers or molecular mechanisms involved in the effects of nutrients deserve special attention too. This Special Issue includes five articles which focus on diet and particular challenges, such as prevalent diseases (metabolic diseases), critical physiological periods (gestation), the gut–brain axis, the recently defined microbial modulator concept of “postbiotic”, or diet–miRNAs interaction. All of the articles included in this Special Issue are examples of the current scientific literature in terms of “Advances in Nutrition”, and they highlight the “Opportunities and Challenges” which have been approached throughout 2022.
